# A systematic review and meta-analysis of the traumatogenic phenotype hypothesis of psychosis

**DOI:** 10.1192/bjo.2024.52

**Published:** 2024-09-09

**Authors:** Franca Onyeama, Eirini Melegkovits, Nicole Yu, Ameerah Parvez, Artur Rodrigues, Jo Billings, Ian Kelleher, Mary Cannon, Michael A. P. Bloomfield

**Affiliations:** Division of Psychiatry, University College London, UK; and Translational Psychiatry Research Group, Research Department of Mental Health Neuroscience, Institute of Mental Health, Faculty of Brain Sciences, Division of Psychiatry, University College London, UK; Translational Psychiatry Research Group, Research Department of Mental Health Neuroscience, Institute of Mental Health, Faculty of Brain Sciences, Division of Psychiatry, University College London, UK; Translational Psychiatry Research Group, Research Department of Mental Health Neuroscience, Institute of Mental Health, Faculty of Brain Sciences, Division of Psychiatry, University College London, UK; and UCL Medical School, University College London, UK; Translational Psychiatry Research Group, Research Department of Mental Health Neuroscience, Institute of Mental Health, Faculty of Brain Sciences, Division of Psychiatry, University College London, UK; and Department of Biology, Faculdade de Ciências da Universidade do Porto, Portugal; Division of Psychiatry, University College London, UK; School of Medicine and Medical Sciences, University College Dublin, Ireland; Department of Psychiatry, Royal College of Surgeons in Ireland, University of Medicine and Health Sciences, Ireland; and Lucena Clinic, St John of God Community Mental Health Services, Dublin, Ireland; Department of Psychiatry, Royal College of Surgeons in Ireland, University of Medicine and Health Sciences, Dublin, Ireland; Translational Psychiatry Research Group, Research Department of Mental Health Neuroscience, Institute of Mental Health, Faculty of Brain Sciences, Division of Psychiatry, University College London, UK; Traumatic Stress Clinic, St Pancras Hospital, Camden and Islington NHS Foundation Trust, London, UK; National Institute for Health Research, University College London Hospitals Biomedical Research Centre, London, UK; and National Hospital for Neurology and Neurosurgery, University College London Hospitals NHS Foundation Trust, London, UK

**Keywords:** Trauma and stressor-related disorders, schizophrenia, childhood experience, phenomenology, psychosis

## Abstract

**Background:**

Developmental trauma increases psychosis risk and is associated with poor prognosis. It has been proposed that psychosis in survivors of developmental trauma gives rise to a distinct ‘traumatogenic’ phenotype.

**Aims:**

Given the implications for personalised treatment, we sought to explore the traumatogenic psychosis phenotype hypothesis in a systematic review and meta-analysis of studies comparing psychotic presentations between adults with and without developmental trauma histories.

**Method:**

We registered the systematic review on PROSPERO (CRD42019131245) and systematically searched EMBASE, Medline and PsycINFO. The outcomes of interests were quantitative and qualitative comparisons in psychotic symptom expression (positive, negative, cognitive) and other domains of psychopathology, including affect regulation, sleep, depression and anxiety, between adults with and without experience of developmental trauma.

**Results:**

Of 34 studies included (*N* = 13 150), 11 were meta-analysed (*n* = 2842). A significant relationship was found between developmental trauma and increased symptom severity for positive (Hedge's *g* = 0.27; 95% CI 0.10–0.44; *P* = 0.002), but not negative symptoms (Hedge's *g* = 0.13; 95% CI −0.04 to 0.30; *P* = 0.14). Developmental trauma was associated with greater neurocognitive, specifically executive, deficits, as well as poorer affect, dissociation and social cognition. Furthermore, psychotic symptom content thematically related to traumatic memories in survivors of developmental trauma.

**Conclusions:**

Our findings that developmental trauma is associated with more severe positive and affective symptoms, and qualitative differences in symptom expression, support the notion that there may be a traumatogenic psychosis phenotype. However, underdiagnosis of post-traumatic stress disorder may also explain some of these findings. More research is needed to explore this further.

Developmental trauma describes experiences of sexual, emotional and physical abuse, and neglect in childhood and adolescence (age <18 years). Considerable evidence suggests that developmental trauma is a risk factor across the psychosis spectrum, including psychotic experiences, at-risk mental states and psychotic disorder.^1^ Developmental trauma is associated with a twofold increase in the likelihood of developing psychosis, and may account for a third of cases of psychosis.^2^ Furthermore, there is evidence of a causal relationship between developmental trauma and psychosis, including clear temporal sequences between exposure and outcome, plausible mechanisms and dose–response relationships.^3,6^ Adults with psychosis and a history of developmental trauma have greater symptom persistence and severity,^7,9^ lower remission rates^10^ and poorer treatment response^11^ compared with those with psychosis who have not experienced developmental trauma. Higher levels of disorganisation^12^ and negative symptoms^8,13,15^ have been reported in individuals who have endured neglect. There is also evidence of differences in hallucinatory content that relates to exposure to developmental trauma, particularly following instances of childhood sexual abuse (CSA).^16^ Elevated post-traumatic stress disorder (PTSD) rates in individuals with psychosis have been observed, as well as a noteworthy phenomenological overlap between PTSD and psychosis symptoms, such as post-traumatic intrusions and hallucinations. The observed parallels and high comorbidity between psychotic symptoms and PTSD have prompted the suggestion that these may constitute a ‘continuum of reactions to trauma’. Additionally, certain cases featuring concurrent PTSD and psychosis symptoms have been conceptualized as ‘PTSD with secondary psychotic features’, a separate ‘disease' entity wherein the absence of formal thought disorder is emphasised.^17^

From a neurodevelopmental perspective, various biopsychosocial processes have been proposed to induce vulnerability to psychosis in survivors of development trauma,^18^ encompassing multiple affective, neurocognitive and behavioural developmental processes.^18,21^ These include, but are not limited to, memory intrusions,^22^ cognitions about the self and others,^18^ and changes in ways of coping with difficult internal and external events post-trauma, as well as emotion regulation and dissociative symptoms.^5,18^ Adopting the perspective of the latent vulnerability theory,^23^ these neurocognitive and behavioural changes can be understood as potentially adaptive responses in the context of maltreating and neglectful environments, which can become maladaptive and contribute to the emergence of psychotic manifestations in the long term.

Although recently it has been repeatedly documented that trauma and psychosis are closely interlinked, our understanding of the phenomenology involved in psychosis in adult survivors of developmental trauma and how this translates in clinical contexts is limited,^24,25^ resulting in a lack of evidence on tailored treatments for this group, with many interventions not accounting for developmental trauma exposure.^25,26^ Furthermore, experiences of developmental trauma are often not considered in adult mental health services, and even less so in individuals with a psychosis diagnosis,^27^ demonstrating that the potential influence of developmental trauma on illness trajectory remains overlooked in the clinic.

Evidence of the higher epidemiological vulnerability to psychosis, poorer prognostic outcomes, and different phenomenological experiences and comorbidities (including, but not limited to, higher post-traumatic stress symptoms) in developmental trauma survivors is suggestive of a trauma-related subtype of psychosis that has been referred to as a ‘traumatogenic’ psychosis phenotype,^28^ i.e. the existence of a clinical phenotype influenced by the dynamic biopsychosocial effects of experiencing psychological trauma. Exploring whether there is a traumatogenic psychosis phenotype is a critical step in the development and future improvements of treatments. When considering the existence of a clinical phenotype, principles of diagnostic validity and clinical utility need to be considered.^29^ These principles encompass having an outline of clinical descriptions (e.g. symptoms) with clear distinctions from other disorders, to provide ease in communication between practitioners for treatment and clinical management.^30,31^ Support of these distinctions would provide preliminary evidence of a traumatogenic psychosis phenotype.

## Aims of the study

Previous studies have explored the strength of the association between trauma and psychosis,^8^ and mechanisms acting as mediators in their relationship,^18^ but no study to date has systematically explored and meta-analytically tested the phenomenological differences in the profiles of patients with psychosis with experiences of maltreatment or neglect in childhood. We sought to explore the presence of a traumatogenic psychosis phenotype by assessing whether psychotic symptoms differ between individuals who have or have not experienced developmental trauma. We separately included studies investigating quantitative (symptom severity) and qualitative (e.g. hallucinatory content) symptoms and phenomenological experiences. Primary outcomes included positive, negative and/or cognitive symptoms (including cognitive outcomes, e.g. working memory). Secondary outcomes were quantitative and/or qualitative differences in other domains of psychopathology and well-being known to be affected in the context of psychosis, such as measures of disorganisation, mood, affect regulation, sleep and anxiety. We then conducted a systematic review and meta-analysis comparing psychosis symptom profiles between adults with and without experience of developmental trauma, synthesising both quantitative and qualitative studies.

## Method

We registered the review with the International Prospective Register of Systematic Reviews (PROSPERO) (identifier CRD42019131245) and followed the Preferred Reporting Items for Systematic reviews and Meta-Analyses (PRISMA) framework.

### Database search

We systematically searched EMBASE, Medline and PsycINFO databases, with no restriction on publication date. Full-text search terms relevant to childhood, psychosis and phenomenology were used (Supplementary materials available at https://doi.org/10.1192/bjo.2024.52). We hand-searched reference lists of relevant studies, OpenGrey and Google Scholar to identify relevant studies. The first search was conducted in April 2019, with subsequent searches in March 2020 and April 2022.

### Eligibility criteria

We used broad inclusion criteria to capture relevant literature. Included studies were quantitative and/or qualitative studies conducted in any country, comparing clinical presentations of psychosis between adults of any gender and ethnicity, with and without histories of developmental trauma and/or who have high versus low exposure to developmental trauma. We defined psychosis to include individuals diagnosed with a psychotic disorder or who presented with psychosis, including at-risk mental states, psychotic symptoms and/or experiences. Symptom presence was determined by diagnostic classification, clinician report or validated outcome measure. We defined developmental trauma as sexual, emotional and physical abuse, and neglect in childhood and adolescence (age <18 years). Presence of developmental trauma was determined through validated outcome measures, self-report measures or reports of childhood maltreatment. Conference proceedings, supplements and studies not comparing groups with exposure to developmental trauma against those without were excluded.

Designs included cross-sectional studies, prospective cohort studies, intervention studies and case–control studies of individuals with and without psychosis, given that they also compared individuals with and without developmental trauma experiences in each group.

### Outcomes

The primary outcomes were quantitative and/or qualitative differences in positive, negative and/or cognitive symptoms (including cognitive outcomes, i.e. working memory) between adults who have and have not experienced developmental trauma. Secondary outcomes were quantitative and/or qualitative differences in other domains of psychopathology and well-being known to be affected in the context of psychosis, such as measures of mood, disorganisation, affect regulation, sleep and anxiety.

### Study selection

Titles and abstracts were screened for eligibility by two independent reviewers in the original and updated searches, and discrepancies were resolved through discussion with a third reviewer. Eligible studies were then screened in full text. Cohen's kappa was used to assess interrater agreement.

### Data extraction

Extracted information (first extracted 5 June 2019) included: sample size, number and size of groups in sample, diagnostic characteristics of sample, measures of psychotic symptoms, developmental trauma type, developmental trauma measure (if applicable), primary outcomes and secondary outcomes. Authors of eligible studies with missing data were contacted. Failure to provide missing information of key variables resulted in study exclusion.

### Risk of bias, quality assessment and level of evidence

We conducted risk of bias (ROB) and quality assessments of quantitative studies using the Newcastle–Ottawa Scale.^32,33^ Scores of 0–3 indicated a high ROB, 4–6 indicated a moderate ROB and 7–10 indicated a low ROB. Qualitative studies were evaluated with the ‘Critical Appraisal Skill Program’ from the Oxford Centre for Evidence-based Medicine.^34^ ROB assessments were conducted independently by two reviewers, and discrepancies were resolved through discussion with another reviewer. Studies with high ROB ratings were assessed with caution, but not excluded. Each study was assigned a ‘level of evidence’ as determined by the Oxford Centre for Evidence-based Medicine.^35^

### Narrative synthesis and content analysis

We conducted a narrative synthesis of quantitative studies, and a content analysis of qualitative data. Descriptions of symptom content and/or meaning were thematically grouped by two reviewers, piloted in one study and subsequently conducted for all studies.

### Meta-analysis

We performed a random-effects meta-analysis^36^ with Stata, version 17 for Windows,^37^ for studies that utilised the same instrument to assess psychotic symptoms. Studies reporting only subcomponent scores of a measure were excluded. We computed Hedge's *g* effect sizes, analysed effect estimates and assessed heterogeneity with the *Q* test. The *I^2^* statistic presented the proportion of variance accounted by heterogeneity.^38^ A significance level of *P* = 0.05 was used. An Egger's test was conducted to test for publication bias.^39^

## Results

### Study characteristics

**Fig. 1:**
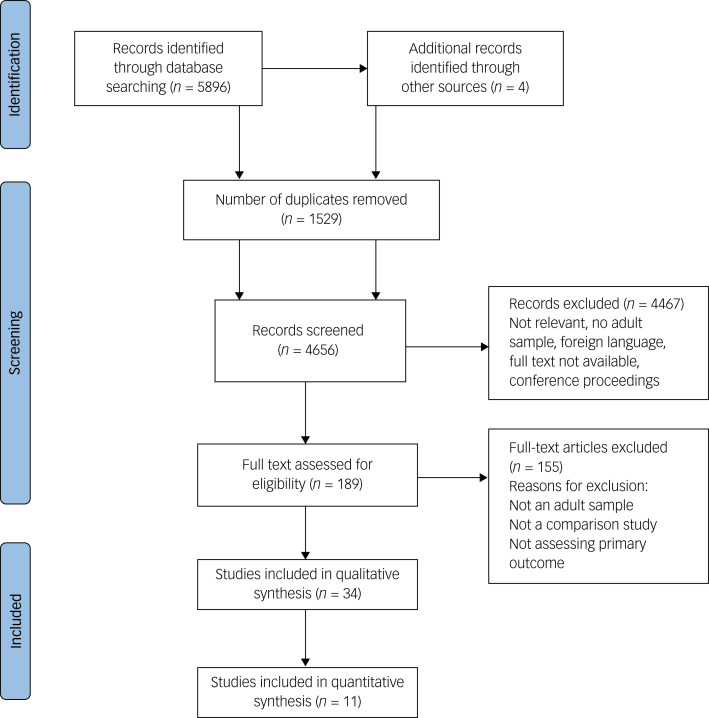
Preferred Reporting Items for Systematic reviews and Meta-Analyses (PRISMA) flow chart.

Details of the search and selection process are presented in a PRISMA flow chart in Fig. 1. Thirty-four studies were included (*N* = 13 150), published between 2001 and 2022. Eleven studies were quantitatively summarised. Good interrater agreement was found for title and abstract, and full-text screening, in the original and updated search (title and abstract: *κ* = 0.78 and 0.696, respectively; full-text: *κ* = 0.74 and 0.620, respectively). Thirty-three studies were quantitative and one study was qualitative.

Ascertainment of exposure to developmental trauma was determined through validated measures in 25 studies, and by self-report/chart reviews in nine studies. Twenty-seven studies compared individuals with and without developmental trauma experiences, and seven studies compared high and low levels of developmental trauma. The majority of studies included individuals with a clinical diagnosis of psychosis. Three studies investigated psychosis in the context of bipolar disorder, and three in the context of psychotic depression. Only five studies^40,44^ explored psychotic symptoms in the general population, and one also in siblings of individuals with psychosis.^45^

### Study design, ROB and level of evidence assessment

ROB and quality assessments are outlined in Tables 1–3. Seven studies were prospective cohort studies and represented the highest level of evidence, 20 studies were cross-sectional and seven studies included a control group. No studies were excluded from the meta-analyses based on ROB. ROB scores ranged from 3 to 9 (mean 5.66, s.d. = 1.537). Of the quantitative studies, 11 were of low ROB, 17 were of moderate ROB and five were of high ROB. The least met criteria were descriptions of comparability between respondents and non-respondents, representativeness of sample and ascertainment of outcome measure.

**Table 1:** Risk-of-bias assessments for case–control studies

Case–control (retrospective) studies	Selection				Comparability	Exposure			Risk of bias
	Adequacy of case definition	Representativeness of the cases	Selection of controls	Definition of controls	Cases and controls matched for confounders	Ascertainment of exposure	Method for case and control ascertainment	Non-response rate	
McCabe et al (2012)^46^	*	*	*	*	**		*		Low
Ayesa-Arriola (2020)^47^	*	*	*	*	**		*		Low
Quidé et al (2018)^48^	*	*	*	*			*		Moderate
Schalinski et al (2019)^49^	*	*	*	*	*		*		Moderate
Chatziioannidis et al (2019)^50^	*	*	*	*	*		*		Moderate

*Met criteria. A maximum of one star is awarded for each item within Selection and Exposure, a maximum of two can be awarded for Comparability and Outcome (**multiple important confounders controlled for).

**Table 2:** Risk-of-bias assessments for cross-sectional studies

Cross-sectional studies	Selection			Comparability	Outcome			Risk of bias
	Representativeness of the sample	Sample size	Comparability between respondents and non-respondents	Ascertainment of exposure	Confounding factors controlled	Assessment of outcome	Statistical test	
Mohammadzadeh et al (2019)^56^	*			**	*		*	Moderate
Lamela and Figueiredo (2018)^44^	*	*	*	**	**	*		Low
Misiak et al (2016)^55^		*		**	**			Moderate
Kelly et al (2016)^51^				**		*		High
Lysaker et al (2001)^57^				*	**	*		Moderate
Shannon et al (2011)^58^		*		**	**			Moderate
Aas et al (2017)^59^				**	*			High
Dorahy et al (2009)^60^				**		*		High
Offen et al (2003)^61^		*		*		*		High
Hardy et al (2005)^62^	*	*		*		**	*	Moderate
Anglin et al (2019)^40^	*	*		**	*	*	*	Low
Schenkel et al (2005)^63^								High
Tosato et al (2020)^64^	*	*		**	*	*		Moderate
Velikonja et al (2019)^65^	*	*		**	*	*	*	Low
Cui et al (2019)^66^		*		**		*	*	Moderate
Comacchio et al (2019)^52^	*	*	*	**				Moderate
Read et al (2003)^67^		*				**	*	Moderate
Laskemoen et al (2021)^68^	*			**	*	**	*	Low
Begemann et al (2021)^43^	*			**	**	**		Low
Mørkved et al (2020)^69^	*			**	**	**		Low
Brañas et al (2022)^70^	*				*	*	*	Moderate

*Met criteria. A maximum of one star is awarded for each item within Selection and Exposure, a maximum of two can be awarded for Comparability and Outcome (**multiple important confounders controlled for).

**Table 3:** Risk-of-bias assessments for prospective cohort studies

Prospective cohort studies	Selection				Comparability	Outcome			Risk of bias
	Representativeness of exposed cohort	Selection of non-exposed cohort	Ascertainment of exposure	Outcome not present at start of study	Exposed group and controls matched for confounders	Assessment of outcome	Follow-up long enough for outcomes to occur	Adequacy of follow-up of cohorts	
Davidson et al (2009)^71^	*	*	*		**		*	*	Low
Schalinski et al (2015)^54^		*	*		**		*	*	Moderate
Lysaker et al (2005)^53^		*			**		*	*	Moderate
Boden et al (2016)^41^	*	*			**		*	*	Moderate
Bell et al (2019)^42^	*	*		*	**		*	*	Low
Van Dam et al (2015)^45^	*	*	*		**		*	*	Low
Kilian et al (2019)^72^	*	*			**		*	*	Moderate

*Met criteria. A maximum of one star is awarded for each item within Selection and Exposure, a maximum of two can be awarded for Comparability and Outcome (**multiple important confounders controlled for).

### Narrative synthesis and meta-analysis of quantitative studies

Two studies identified gender differences. One study, not accounting for confounders,^51^ observed a gender difference whereby females with developmental trauma exposure reported significantly more positive symptoms than men with developmental trauma, as well as males with and without developmental trauma experiences. Another study identified a gender difference whereby there was a decrease in the age at onset of first-episode psychosis in females with a history of physical or sexual trauma compared with males with similar experiences, or males or females with no developmental trauma experiences.^52^

Mixed findings emerged regarding the impact of developmental trauma on prognosis: one low ROB, well-conducted, prospective cohort study demonstrated that those who had experienced abuse had significantly higher scores on the positive symptoms scale as assessed biweekly over 16 weeks;^48^ however, another low ROB prospective study did not find differences in positive or negative symptoms over a 3-year period.^45^ Interestingly, Schalinski and colleagues^54^ recorded an overall reduction of symptoms, as measured by the Positive and Negative Syndrome Scale (PANSS), over a 4-month period in an in-patient sample for both those with and without developmental trauma experiences, but no association emerged between symptom severity course and developmental trauma status. However, severity of developmental trauma was positively related to drop-out and hospital readmission.

Medication information was inconsistently reported, with 21 studies not reporting any information on specific medication prescribed, and some studies providing broad descriptions such as ‘antipsychotics’, ‘antidepressants’, ‘mood stabilisers’ and ‘neuroleptics’. Additionally, only seven studies accounted for substance and alcohol misuse.

### Primary outcome

#### Positive symptoms

**Fig. 2:**
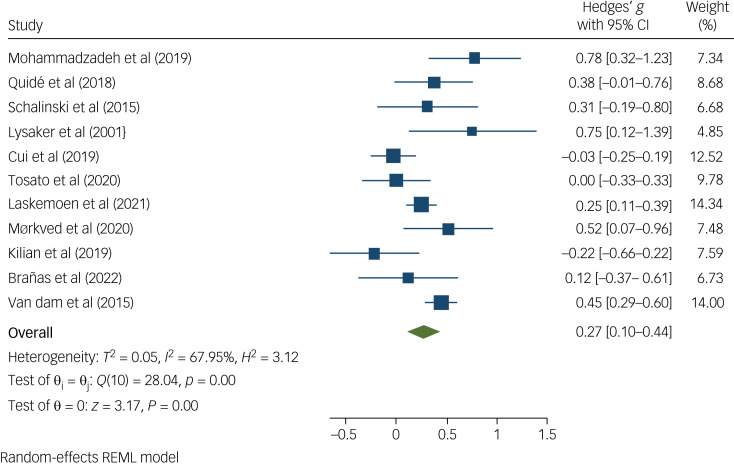
Forest plot showing the standardised mean difference in positive symptom score between people with and without developmental trauma exposure. REML, restricted maximum likelihood.

Nineteen studies – eight of low, ten of moderate and one of high ROB (Supplementary Table 1) – assessed differences in total positive symptoms in those with and without developmental trauma histories. Of these, four were of a high level of evidence. The meta-analysis indicated that individuals with psychosis and developmental trauma experiences scored significantly higher on the PANSS positive subscale than those without developmental trauma experiences (Hedge's *g* = 0.27; 95% CI 0.10–0.44; *P* = 0.002) (Fig. 2). Heterogeneity was statistically significant (*I^2^* = 67.95%, *P* = 0.002), and Egger's regression was not statistically significant (*P* = 0.462). Findings from studies not included in the meta-analysis also reflected this relationship. Of the nine studies excluded from the meta-analysis, six identified higher positive symptoms in participants with developmental trauma exposure, compared with those without (Supplementary Table 1). Greater symptom severity followed greater and lengthier trauma exposure, multiple forms of abuse^50^ and sustaining physical injuries.^44^

#### Hallucinations

Five studies assessed the relationship between developmental trauma and hallucinations (Supplementary Table 1) quantitatively. Across studies, participants with psychosis and experience of developmental trauma scored higher on measures of hallucinations than those without.^50,53,55^ Severity and frequency of developmental trauma was associated with increased hallucination severity, and a dose–response effect between developmental trauma and hallucinations emerged.^63^ Another study reported more first-rank auditory verbal hallucinations (running commentary, third-person auditory hallucinations and thought echo) and more hallucination categories (i.e. abusive, accusatory and persecutory voices) in first episode psychosis participants with developmental trauma experiences than in those without. Hallucinations were positively associated with specific abuse types, namely maternal neglect and antipathy (i.e. hostile/rejecting behaviours toward the child),^50,55^ and with having sustained physical injuries,^37^ and their controllability was associated with emotional developmental trauma.^43^

#### Delusions

Three studies reported on delusion severity. Although one study^63^ of low ROB found a significant difference between groups, two separate studies^53,67^ of moderate ROB and a high level of evidence found no differences in delusions or suspiciousness between those with and without developmental trauma experiences.

#### Negative symptoms

Twenty-two studies investigated negative symptoms (Supplementary Table 1). Eight studies were of low, 11 were of moderate and three were of high ROB, with shared methodological issues, including a lack of blind assessment and comparability between respondents and non-respondents.

**Fig. 3:**
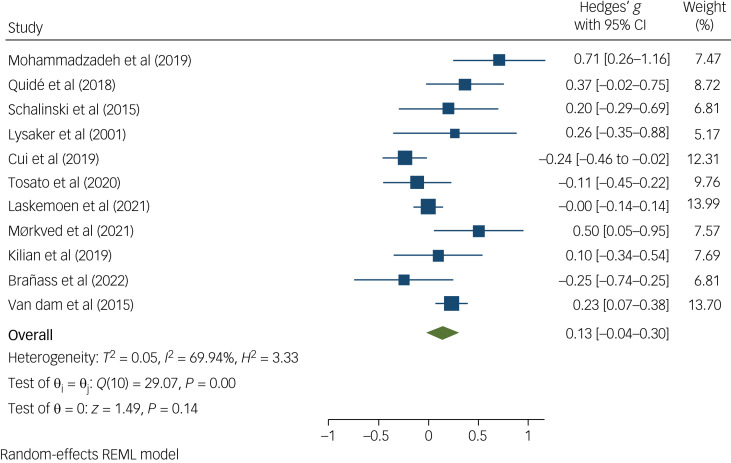
Forest plot showing the standardised mean difference in negative symptom score between people with and without developmental trauma exposure. REML, restricted maximum likelihood.

The meta-analysis revealed no significant difference in scores on the PANSS negative subscale between groups (Hedge's *g* = 0.13; 95% CI −0.04 to 0.30; *P* = 0.14) (Fig. 3). Of the 11 studies not included in the meta-analysis, only one study found differences, wherein survivors of physical and sexual developmental trauma had significantly higher PANSS negative subscale scores than controls (*P* < 0.01).^52^

#### Cognition

Nine studies assessed the relationship between cognitive outcomes and developmental trauma in individuals with psychosis (Supplementary Table 1). Two studies identified worse general cognitive function,^47,69^ particularly associated with physical neglect.^69^ Six studies found reduced working memory performance.^47,53,57,58,65,69^ Mixed findings emerged from two studies^53,69^ measuring verbal memory with the California Verbal Learning Test: Mørkved and colleagues^69^ reported worse performance in individuals with schizophrenia spectrum disorders and developmental trauma experiences than those without developmental trauma, whereas Lysaker and colleagues^57^ reported no between-groups difference. Poorer performance in verbal learning fluency was associated with severity of trauma among individuals with schizotypal personality disorder in a low ROB study.^65^ Three studies did not identify any differences in cognitive performance in participants with psychosis and developmental trauma compared with those without developmental trauma exposure: one of high ROB,^51^ one of moderate ROB^50^ and one of low ROB.^46^ Interestingly, Chatziioannidis and colleagues^50^ established differences in intellectual functioning associated with developmental trauma only in healthy controls. Finally, two low ROB studies identified reduced performance in visuospatial abilities.^65,69^

### Secondary outcomes

Twelve studies identified that developmental trauma was associated with higher depression and emotional distress. Three of these studies compared depressive symptoms among those with high versus low developmental trauma status. Three studies found no difference in distress and affect^54,64,71^ between groups. Participants with psychosis and a history of high developmental trauma presented with more negative schemata, less positive schemata and more rumination than those with low developmental trauma exposure.^66^

In particular, studies identified that emotional, physical^66^ and sexual abuse,^56,66^ and physical neglect,^56^ were associated with increased depressive symptoms, as well as higher risk of suicide^56^ and lifetime suicidal ideation and attempts.^56,66^ Mixed findings emerged regarding mania,^68^ with two studies of low^61^ and moderate^64^ ROB not establishing differences between groups in levels of mania.

Four studies assessed the association between developmental trauma and dissociation.^40,53,60,61^ Only one study was of low ROB. All studies found higher levels of dissociation in developmental trauma groups.^40,53,60,61^ Dissociation mediated the relationship between developmental trauma and positive psychotic symptoms.^40^ Interestingly, in Anglin and colleagues,^40^ dissociation mediated the relationship between developmental trauma and psychotic experiences only in the high cumulative trauma group, and a dose–response effect emerged between dissociation and psychotic experiences in groups with developmental trauma.

One study exploring differences in sleep between participants with schizophrenia and bipolar disorder with and without developmental trauma exposure, found higher frequency of insomnia but lower frequency of hypersomnia in those with developmental trauma experiences. Furthermore, the four^47,59,66^ studies assessing disorganisation did not establish differences associated with developmental trauma.

Differences were also observed in social cognition, and in particular in facial affect recognition. In a study of high ROB, individuals with high developmental trauma levels reported positive faces as less positive and negative faces as more negative,^59^ higher differentiation of negative faces and greater supramarginal and middle temporal gyrus activation in response to negative faces; however, another study of moderate ROB^70^ failed to replicate this result, only finding greater fear recognition in those who had experienced abuse versus those who had not. Regarding specific types of abuse, those with ‘physical or emotional’ abuse were more likely to rate faces as fearful compared with those with sexual abuse.^70^

### Qualitative content analysis

Six quantitative and one qualitative study were analysed comparing qualities and content of psychotic symptoms between participants with and without developmental trauma.

#### Hallucinations

Seven studies compared the content of auditory hallucinations, whereas three studies compared the type of hallucinations (Supplementary Table 2). Qualitative differences were established in hallucinatory content, phenomenological characteristics and emotional responses to the voices.

In terms of hallucinatory content, individuals with developmental trauma experienced hallucinatory content related to traumatic memories.^43,53,73^ Two studies^53,73^ reported that hallucinatory voices in developmental trauma survivors were related to perpetrators of abuse. Hallucinations were also described as more thematically threatening and negative in those with developmental trauma than controls,^43,53,55,62,73^ with more abusive, accusatory and persecutory voices.^55^

Further, mixed findings emerged regarding the commanding nature of hallucinations; one high^60^ and one moderate^53^ ROB study found trauma-related hallucinations were more commanding in those with developmental trauma, which was not replicated by a low ROB study.^60^ Only one study^71^ with unadjusted confounds found that developmental trauma status did not differentiate the experience of threatening hallucinations.

Phenomenological differences in hallucinations included the loudness and location of voices:^43,60^ the high ROB study of Dorahy and colleagues^60^ found louder, more predominant and less externally experienced voices than normal speech in adult survivors of developmental trauma, but no differences were established by Begemann and colleagues.^43^ Trauma-related elements were also present in the sensory modality of hallucinations, whereby two studies^60,67^ reported olfactory and gustatory, in addition to auditory, hallucinations in those who survived developmental trauma.

Regarding the responses to hallucinations, three studies^43,55,73^ reported that individuals with developmental trauma experiences reported more malevolent and unpleasant voices, and invoking fear and threat of abuse,^55,73^ compared with voices reported by those without developmental trauma histories. Recounts of such malevolent voices included ‘He go, “Oh, I can see you” ( … ) He talk like he going to rape me’.^73^ One study also described verbal hallucinations as more omnipotent in participants with high developmental trauma.^43^

Finally, differences were identified in voices in relation to developmental trauma subtypes. In cases of sexual abuse, compared with other types of abuse, there were more frequent reports of command hallucinations to harm/or kill oneself,^54^ experiencing more malevolent voices, as well as more descriptions of visual (e.g. ‘sees a man standing in room’) and olfactory hallucinations (bad odour in bed at night seeping out) connected to the trauma. Fear of sexual abuse being repeated and voices threatening or alluding to rape (‘want my body’) were also described.^73^ Individuals with CSA experienced voices of perpetrators, with one participant recalling a ‘voice of the relative telling me to jump from the bridge and kill myself’.^54^ In cases of physical abuse, more descriptions of command hallucinations to harm/kill oneself, as well as tactile and visual hallucinations connected to the trauma, were reported. One study identified an association between controllability of voices and emotional developmental trauma, even when compared with high developmental trauma exposure.^43^

#### Delusions

Two well-conducted studies^42,73^ reported comparisons of delusions. Individuals with severe developmental trauma exposure were more commonly reported to experience delusions of persecution or guilt and delusions of reference than those with little or no exposure.^42^ Developmental trauma survivors were also more likely to experience grandiose delusions.^73^

## Discussion

To our knowledge, this is the first review that has addressed the presence of a traumatogenic phenotype of psychosis by assessing and identifying quantitative and qualitative differences in psychotic symptom presentation between individuals with and without a history of developmental trauma. We found evidence of an association between developmental trauma and increased positive and cognitive, but not negative, symptom severity. Developmental trauma was also associated with increased symptom severity in other domains of psychopathology. Some evidence connected positive symptom content with characteristics and themes of traumatic memories in developmental trauma survivors. Our findings provide some evidence in favour of a traumatogenic phenotype of psychosis, and are discussed in the context of methodological strengths and limitations, alternative explanations such as undiagnosed PTSD, and suggestions for future research and clinical practice.

### Symptom severity

#### Positive symptoms

The first preliminary indicator of a traumatogenic phenotype of psychosis may be the symptom severity differences established between adults with and without experiences of developmental trauma.

Importantly, we found evidence of specificity regarding hallucinations, such that hallucination severity related to developmental trauma severity, in keeping with a previous systematic review and meta-analysis.^8^ Still, this finding might simply reflect exposure dose–response effects and also be biased as a result of limited studies having investigated an association between developmental trauma and delusion or paranoia.

Strikingly, we also found that as the course of psychosis unfolds, positive symptoms and outcomes continue to worsen in developmental trauma survivors,^49,53^ and they face increased risk of treatment drop-out and hospital readmission.^53^ This is comparable to findings indicating that the illness course of depression worsens over time in adults who have survived developmental trauma.^69^ Potential explanations for the worsening of symptoms over time could be the inadequate effectiveness of current interventions for individuals with developmental trauma experiences, as well as potential underlying mechanisms maintaining psychotic symptoms, such as a progressive clinical phenotype, PTSD symptoms or dissociation.

#### Negative symptoms and cognition

We found evidence that developmental trauma was associated with greater neurocognitive deficits, specifically executive dysfunction including impaired working memory, supporting a recent review that found greater global (i.e. lower IQ) and specific cognitive deficits^74^ were associated with developmental trauma in psychosis. Developmental trauma could lead to alterations in underpinning neurocognitive systems,^75^ such as impairments in working memory,^76^ autobiographical memory^77^ and social cognition,^72^ which can persist into adulthood^77^ and increase vulnerability to anomalous experiences and psychotic symptom development.^75^ Supporting this, we identified greater fear recognition and accentuation of appraisal of faces as negative in individuals with psychosis who had experienced developmental trauma. Our review supported the link between working memory deficits,^78^ and autobiographical memory deficits and the experience of auditory hallucinations, which may in fact be a form of traumatic memory intrusions,^79,80^ a finding also corroborated by the evidence we identified on the trauma-related content of hallucinations.

We found no differences in negative symptoms associated with developmental trauma, in line with previous research.^8^ Nonetheless, converging evidence,^13,15^ including support from a previous meta-analysis,^8^ delineates a small association between childhood neglect and increased negative symptom severity. One potential explanation for the discrepancy in our findings is that the study by Bailey et al^8^ delved into the associations between severity of developmental trauma and symptoms of psychosis, whereas we reviewed studies that directly compared the clinical presentations of psychosis between adults who have and have not experienced developmental trauma.

### Secondary outcomes

We found moderate evidence of increased symptom severity in depression and anxiety, which is in line with previous research,^81,82^ as well as consistently higher levels of dissociation associated with developmental trauma in patients with psychosis, supporting previous research that dissociation mediates the relationship between developmental trauma and psychotic symptoms.^83^ One putative explanation is that dissociation occurs as a protective response in the context of inescapable danger, but in the long term it becomes a habitual and maladaptive post-traumatic mechanism, which may give rise to hallucinations, paranoia and greater symptom severity.^84,85^ Nevertheless, higher levels of dissociation may reflect undetected PTSD symptoms. No differences emerged in disorganisation, although few studies explored this outcome.

### Qualitative differences

The content of auditory hallucinations in developmental trauma survivors reflected characteristics and themes of their traumatic events. This is suggestive of schematised material,^86^ such as the experience of symbolised manifestations of trauma-related content in both the thematic content^62^ and quality of hallucinations.^67,73^ Regarding hallucination content, research also demonstrated that past trauma is symbolically reflected in hallucinatory content in individuals with psychosis,^87^ and that individuals who experienced CSA may present with psychotic symptoms with sexual content.^88^ This demonstrates the importance of how experiencing trauma may create a ‘trauma lens’, through which meaning is later constructed. For example, individuals with religious backgrounds may tend to have more religious content in their psychotic symptoms.^89^

Furthermore, developmental trauma survivors also reported experiencing more abusive and persecutory voices.^73^ These were reported to be fear-invoking, which could reflect an association to threatening trauma memories, e.g. the re-experiencing of a perpetrator's voice giving rise to the emotion of fear. Importantly, this may also be suggestive of a re-experience of trauma memories, which is a core symptom of PTSD.^90^

### Clinical phenotype or undiagnosed PTSD?

Taken together, we found evidence of quantitative differences in symptom profiles based on exposure to trauma, wherein adult survivors of developmental trauma presented with greater symptom severity across positive symptoms, and hallucinations in particular; worse working memory performance; difficulties with mood and affective processes, such as depression and dissociation; and poorer prognostic outcomes. The evidence supporting this was good, with most studies of low to moderate ROB. We also found qualitative differences in symptom expression and phenomenology, with manifestations of trauma-related memory content in those with developmental trauma experiences. This supports the hypothesis that individuals with developmental trauma experiences may present with a traumatogenic psychosis phenotype, with phenomenologically different and more severe psychotic symptoms than those without developmental trauma exposure. It should be noted that some of the findings might be accounted for by reverse causality, considering the potential neurocognitive and affective burden of psychotic illness.

A complementary and clinically parsimonious interpretation of the traumatogenic psychosis phenotype is that findings in some patients may be suggestive of a psychotic form of PTSD.^17^ We have recently reported that psychological processes associated with PTSD underlie the relationship between developmental trauma and psychosis.^18^ Adult survivors of developmental trauma have increased risk of developing PTSD,^91^ including intrusive memories and feeling threatened ‘in the here and now’,^92^ emotional dysregulation, functional impairments^93^ and psychotic symptoms.^94^ Furthermore, traumatic re-experiencing seen in hallucinatory content could be accounted for by information-processing models of PTSD, suggesting that deficits in contextual integration result in the intrusion of unintended trauma-related memories, which may occur as voices.^22,90,95,96^ The impairments in emotion dysregulation identified in our study, namely dissociation, could be a manifestation of PTSD^97,99^ and worsen the severity of symptoms in patients with psychosis and developmental trauma experiences.

Alternatively, given the striking absence of measurement of PTSD in included studies, we cannot exclude the possibility that undiagnosed PTSD or PTSD symptoms, comorbid to schizophrenia spectrum disorders, could account for some of our findings. PTSD is often underdiagnosed and/or misdiagnosed in patients, especially those with psychotic symptoms,^100,101^ and is associated with poorer outcomes and lack of appropriate and effective treatment when undiagnosed.^102,103^ Additionally, as posited in a seminal paper, psychosis can be a traumatic event inducing PTSD responses, but psychosis can also emerge as a reaction to trauma.^104^ Although we acknowledge the correlation and overlap between PTSD and psychosis, developmental trauma seems to lead to a synergistic interplay of affective, neurocognitive and behavioural processes that contribute to a phenomenologically distinct and more severe presentation, with symptoms that extend beyond PTSD, in what we propose as a traumatogenic psychosis phenotype.

### Strengths and limitations

Key strengths of the review include the use of broad inclusion criteria to identify relevant literature, the use of the PRISMA guidelines and the inclusion of both quantitative and qualitative findings, through the methods of narrative synthesis and meta-analysis. This allowed us to paint a holistic picture of the possible impact of developmental trauma, by studying differences along the psychosis continuum and the value of information on both symptoms and phenomenology/subjective experience.

We encountered several methodological limitations in included studies. First, most studies failed to compare between participants who did and did not participate. Most included studies were cross-sectional, limiting causal inferences,^105^ and retrospectively measured developmental trauma, introducing potential recall bias or error.^106^ Also, most studies did not study psychotic symptoms in relation to PTSD, a common consequence of developmental trauma, limiting our understanding of the nature of a distinct clinical traumatogenic psychosis phenotype. Finally, the majority of studies did not include sufficient information on medication status, potentially biasing findings on the clinical presentation of participants.

Regarding limitations of the present study, inductive bias may have influenced the conceptual approach taken, where a direct causal association between developmental trauma and psychosis was assumed. A further limitation relates to the small number of studies included in the meta-analysis; a low number of pooled studies may result in weaker power of the overall effect size estimate.^107^ Finally, it should be added that developmental trauma is only one of many factors known to predict psychosis, such as a biological predisposition, prenatal and perinatal factors, substance misuse, as well as other forms of early adversity and traumatic events further in life, which this study did not account for.

### Suggestions for future research

Regarding aetiological factors, evidence emerged on neurocognitive differences associated with a history of developmental trauma, rendering the currently limited explorations of the biological mechanisms underpinning the relationship between developmental trauma and psychosis necessary. Further research into the relationship between trauma memories and hallucinations is needed, and whether a diagnostic construct of psychotic PTSD^17^ offers clinical validity, parsimony and utility to inform interventions. It is also imperative to understand how experiences of developmental trauma interact with other biological, psychological, socioeconomic and cultural factors in predicting emergence and/or clinical outcomes in psychosis.

Most studies were limited on the relationship between developmental trauma and positive and negative symptoms broadly, as well as hallucinations, with little description of other key aspects of symptoms in psychosis. This highlights the importance of further research to deepen our understanding of the total potential impact of developmental trauma on psychosis, including delusions, negative symptoms, disorganisation, affective regulation and sleep.

Although our study adds to a body of research that highlights the augmented needs of individuals with psychosis and developmental trauma, a recent meta-analysis^18^ suggested that evidence on the relative effectiveness of different treatments for this population is limited and weak.^18^ Considering the poor treatment prognosis in survivors of developmental trauma with psychosis, it is necessary to develop and test the feasibility and effectiveness of interventions that address the additional needs of this group, informed by clinician and patient participation and measuring adverse effects. Treatment components could involve addressing emotion regulation, dissociation and themes related to developmental trauma in the content (e.g. voices of perpetrators, particular sensations), experience, appraisal and response to hallucinations (e.g. malevolence, threat, controllability, avoidance/suppression). In recent years, various trials have commenced that address this gap in treatment. The ‘Talking with Voices’ method^85,108,109^ is an illustration of a treatment approach that aims to re-evaluate the relationship between voice hearers and their voices, and integrate what is viewed as a ‘dissociated’ aspect of experience, potentially linked to trauma, that manifests through hallucinations.

Emerging evidence suggests that trauma-focused psychotherapies for individuals with PTSD who experience psychosis may be effective,^110^ with a range of trials ongoing. Notably, the majority of trauma-focused trials on psychosis focus on individuals with comorbid PTSD diagnoses, thus excluding a large portion of people with psychosis and histories of developmental trauma. Examples include the RE.PROCESS trial,^111^ a multicentre randomised controlled trial that will compare the effectiveness of three trauma-focused treatment modalities (cognitive restructuring, prolonged exposure and eye movement desensitisation and reprocessing) in addition to treatment as usual for psychosis; and the STAR trial, which will compare trauma-focused cognitive–behavioural therapy for psychosis in addition to treatment as usual versus treatment as usual alone.^112^ A recent feasibility, randomised controlled trial also identified eye movement desensitisation and reprocessing for psychosis as a feasible and promising approach for adults with experiences of developmental trauma in early intervention in psychosis settings, including for those who do not have a PTSD diagnosis.^113^

Overall, although initial findings are promising, research of high methodological rigor is needed to explore the potential differential response to current and new evidence-based treatments in this population.^18^ In addition, future work could explore whether there are differential responses to treatment in patients with PTSD with psychosis versus developmental trauma survivors who experience psychosis without PTSD.

### Clinical implications

Clinical practice recommendations are discussed through the GRADE (Grading of Recommendations, Assessment, Development and Evaluations) framework.^114^ The symptom differences identified in those with psychosis and developmental trauma experiences through our meta-analysis, narrative synthesis and qualitative content analysis carry important implications for clinical practice. First, we found strong evidence of an association between developmental trauma and positive symptoms, which was replicated consistently in the majority of studies, included a dose–response effect and was not affected by publication bias in our meta-analysis. In view of strong evidence from mostly level 1 and level 2 studies, a grade A recommendation is that there should be a targeted assessment of developmental trauma in patients with positive symptoms, which should be incorporated in the delivery of treatment. Second, if developmental trauma is identified, the presence of trauma-related content and themes associated with the experience of trauma in symptoms, namely hallucinations, should be explored and considered during formulation and treatment. This should be considered as a grade B recommendation.

Although reviewed studies did not assess for PTSD, considering that PTSD is often underdiagnosed and/or misdiagnosed in patients with psychotic symptoms,^100,101^ we recommend that targeted assessment of PTSD should occur in patients with psychosis and developmental trauma.^114^ This can help clarify psychiatric assessment and inform psychological formulation and intervention, such as whether specialist trauma-focused treatment is warranted.

In conclusion, adult survivors of developmental trauma have greater psychotic symptom severity, including more severe positive and depressive/anxious symptoms, than those without developmental trauma. The content of hallucinations may reflect re-experiencing trauma memories or schematised material deriving from experiences of trauma. Acknowledging and developing a nuanced shared understanding of these phenotypical differences, and the potential commonalities and differences with individuals who experience psychotic PTSD, is crucial for the development of tailored interventions that consider the multifaceted impact of developmental trauma. Further longitudinal research is needed to disentangle these relationships.

## Supporting information

Onyeama et al. supplementary material Onyeama et al. supplementary material

## Data Availability

Data availability is not applicable to this article as no new data were created or analysed in this study.
