# Supplementary material for: A systematic review and meta-analysis of the traumatogenic phenotype hypothesis of psychosis
Source: BJPsych Open. 2024 Sep 9;10(5):e146. doi: 10.1192/bjo.2024.52 (PMC11698141; doi:10.1192/bjo.2024.52)
Supplement: Onyeama et al. supplementary material [file S2056472424000528sup001.docx]

**Supplementary materials**

**PRISMA-P (Preferred Reporting Items for Systematic review and Meta-Analysis Protocols) 2015 checklist: recommended items to address in a systematic review protocol***

| Section and topic | Item No | Checklist item | | |  | | | Page | |  |  |
| --- | --- | --- | --- | --- | --- | --- | --- | --- | --- | --- | --- |
| ADMINISTRATIVE INFORMATION | | | |  | |  | | | |  |  |
| Title: |  | |  | | | |  | |  |  |  |
| Identification | 1a | Identify the report as a protocol of a systematic review | | |  | | | Page 2 | |  |  |
| Update | 1b | If the protocol is for an update of a previous systematic review, identify as such | | |  | | | N/A | |  |  |
| Registration | 2 | If registered, provide the name of the registry (such as PROSPERO) and registration number | | |  | | | Page 2 | |  |  |
| Authors: |  |  | | |  | | |  | |  |  |
| Contact | 3a | Provide name, institutional affiliation, e-mail address of all protocol authors; provide physical mailing address of corresponding author | | |  | | | Page 1 | |  |  |
| Contributions | 3b | Describe contributions of protocol authors and identify the guarantor of the review | | |  | | | Page 23 | |  |  |
| Amendments | 4 | If the protocol represents an amendment of a previously completed or published protocol, identify as such and list changes; otherwise, state plan for documenting important protocol amendments | | |  | | | N/A | |  |  |
| Support: |  |  | | |  | | |  | |  |  |
| Sources | 5a | Indicate sources of financial or other support for the review | | |  | | | Page 22 | |  |  |
| Sponsor | 5b | Provide name for the review funder and/or sponsor | | |  | | | N/A | |  |  |
| Role of sponsor or funder | 5c | Describe roles of funder(s), sponsor(s), and/or institution(s), if any, in developing the protocol | | |  | | | N/A | |  |  |
| INTRODUCTION | | | |  | | | | | | |  |
| Rationale | 6 | Describe the rationale for the review in the context of what is already known | | |  | | | Page 4 | |  |  |
| Objectives | 7 | Provide an explicit statement of the question(s) the review will address with reference to participants, interventions, comparators, and outcomes (PICO) | | |  | | | Page 5 | |  |  |
| METHODS | | | |  | | | | | | |  |
| Eligibility criteria | 8 | Specify the study characteristics (such as PICO, study design, setting, time frame) and report characteristics (such as years considered, language, publication status) to be used as criteria for eligibility for the review | | |  | | | Page 6 | |  |  |
| Information sources | 9 | Describe all intended information sources (such as electronic databases, contact with study authors, trial registers or other grey literature sources) with planned dates of coverage | | |  | | | Page 6 | |  |  |
| Search strategy | 10 | Present draft of search strategy to be used for at least one electronic database, including planned limits, such that it could be repeated | | |  | | | N/A | |  |  |
| Study records: |  |  | | |  | | |  | |  |  |
| Data management | 11a | Describe the mechanism(s) that will be used to manage records and data throughout the review | | |  | | | N/A | |  |  |
| Selection process | 11b | State the process that will be used for selecting studies (such as two independent reviewers) through each phase of the review (that is, screening, eligibility and inclusion in meta-analysis) | | |  | | | Page 7 | |  |  |
| Data collection process | 11c | Describe planned method of extracting data from reports (such as piloting forms, done independently, in duplicate), any processes for obtaining and confirming data from investigators | | |  | | | Page 7 | |  |  |
| Data items | 12 | List and define all variables for which data will be sought (such as PICO items, funding sources), any pre-planned data assumptions and simplifications | | |  | | | Page 7 | |  |  |
| Outcomes and prioritization | 13 | List and define all outcomes for which data will be sought, including prioritization of main and additional outcomes, with rationale | | |  | | | Page 6 | |  |  |
| Risk of bias in individual studies | 14 | Describe anticipated methods for assessing risk of bias of individual studies, including whether this will be done at the outcome or study level, or both; state how this information will be used in data synthesis | | |  | | | Page 7 | |  |  |
| Data synthesis | 15a | Describe criteria under which study data will be quantitatively synthesised | | |  | | | Page 7 | |  |  |
|  | 15b | If data are appropriate for quantitative synthesis, describe planned summary measures, methods of handling data and methods of combining data from studies, including any planned exploration of consistency (such as I^2^, Kendall’s τ) | | |  | | | Page 7 | |  |  |
|  | 15c | Describe any proposed additional analyses (such as sensitivity or subgroup analyses, meta-regression) | | |  | | | N/A | |  |  |
|  | 15d | If quantitative synthesis is not appropriate, describe the type of summary planned | | |  | | | Page 7 | |  |  |
| Meta-bias(es) | 16 | Specify any planned assessment of meta-bias(es) (such as publication bias across studies, selective reporting within studies) | | |  | | | Page 7 | |  |  |
| Confidence in cumulative evidence | 17 | Describe how the strength of the body of evidence will be assessed (such as GRADE) | | |  | | | N/A | |  |  |

**Full-text search terms**

1. “Child” OR “Childhood” OR “Young Adult” OR “Young People” OR “Young Person” OR “Adolescen*” OR “Development*” OR “Teenage*”

2. “Psychological Trauma” OR “Emotional Trauma” OR “Abuse” OR “Maltreatment” OR “Neglect” OR “Bully*”

3. “Psychotic” OR “Psychosis” OR “Schizoph*” OR “Schizoa*” OR “Schizotyp*” OR *Non-organic Psychosis” OR “Non-Affective Psychotic Disorder”

4. “Symptom” Or “Symptomatol*” Or “Phenomenol*” Or “Hallucinat*” Or “Delusio*” Or “Thought Disorder” OR “Severity” OR “Qualitat*” OR “Quantita*” OR “Experience” OR “Events”

Table 1. Characteristics of Included Studies

| **Literature** | **Design** | **Evidence**  **level** | **Sample (N of groups, age range or M/SD, diagnoses, medication)** | **Measure of DT and types of DT** | **Primary outcome**  **measure** | **Primary outcome** | **Secondary outcome measure** | **Secondary outcomes** |
| --- | --- | --- | --- | --- | --- | --- | --- | --- |
| Mohammadzadeh et al. (2019) | Cross-sectional | 2b | Total:82  32 high DT  50 low DT  Clinical psychosis  aged 18-59 with SCZ (DSM-IV-TR)  “Antipsychotics not specified”.  Patients were excluded if they had “substance use” | CTQ-SF (≥46 = high DT+)    Emotional, physical and sexual abuse, physical and emotional neglect | PANSS | High DT+ scored higher on PANSS positive and PANSS negative scales than Low DT+    Effect size n/r | BDI-II: depression  BSSI: suicidal ideation | High DT+ scored higher on measures of depression and suicidal ideation than Low DT+    Partial η2 = 0.05 – sexual abuse  Partial η2 = 0.05 - physical neglect) |
| Quidé et al. (2018) | Case-control | 3b | 71 P+DT,  41 P-DT,  21 HC+DT,  32 HC-DT  18-65  Healthy controls and clinical psychosis  aged 18-65 with SCZ, SAD, BD with psychosis  (ICD-10)  “Antipsychotics” and “antidepressants” not specified  Mood Stabilizers (including lithium, carbamazepine, valproate and lamotrigine): N=53 | CTQ-SF    Emotional, physical and sexual abuse, physical and emotional neglect | PANSS | P+DT scored higher on PANSS positive, negative and total subscales than P-DT    Effect size n/r | DASS-21: Depression, anxiety, stress  MADRS: Depression  BDRS: Bipolar symptoms  YMRS: mania | DT+ scored higher on measures of depression, anxiety, and stress, bipolar. and mania than DT-  Effect size n/r |
| Schalinski et. al (2019) | Case-control | 3b | 138 P+DT,  42 P-DT  33 HC+DT  37 HC-DT  28.6 (8.8)  Healthy controls and clinical psychosis with SCZ, Acute Polymorph Psychotic Disorder, SAD, Delusional disorder  (DSM-IV)  “Neuroleptics” not specified= N=172 | LEC; MACE    Physical, verbal/non-verbal emotional abuse.  Emotional and physical neglect; Physical abuse (whipping, slapping/kicking, poking/punching, and hair-pulling) | PANSS | DT+ have more severe positive symptoms than DT-  No difference in negative symptoms between DT+ and DT-      Effect size n/r | Shutdown Dissociation Scale: dissociation | Dissociation mediated the relation of trauma load in childhood trauma on positive symptoms accounting for 25.9% of the total effect. |
| Lamela & Fugueiredo (2018) | Cross-sectional | 2b | 941 (696 DT+)  General population  aged 22-59 with  delusions and  hallucinations  (BSI)  Medication information n/r | CHQ    Physical abuse (whipping, slapping/kicking, poking/punching, and hair-pulling) | BSI: psychoticism (hallucinations and delusions) | DT+ with injuries scored  higher on the psychoticism  dimension compared to DT+  with no injuries and DT-  Effect size n/r | Portuguese BSI:  GSI: severity | DT+ with injuries scored higher on the other BSI dimensions compared to DT+ with no injuries and DT- |
| Kelly et al. (2016) | Cross-sectional | 2b | 80 (21 DT+)  18-75  Clinical psychosis with  SCZ, SAD (DSM-IV)  Medication information  n/r | CTQ    Physical abuse | BPRS:  psychosis  RBANS: cognitive function | Women DT+ had higher scores  on positive symptoms but not  negative symptoms than women  DT- and men with DT+ and DT-  No differences in cognitive  function (RBANS) between  groups | CDS:  depression | Women DT+ had higher scores on  depressive symptoms than women  DT- and men with DT+ and  DT-. |
| Schalinski, Fischer, Rockstroh (2015) | Prospective cohort | 1b | 62 (33 with high DT+)  32.2 (10.3)  Clinical psychosis with SCZ, SAD, Acute Polumorphic Psychotic Disorder  (ICD-10)  “Neuroleptics” not specified  Pt with current substance abuse were excluded. | MACE  (High DT = 2 or more trauma types)    Physical, and emotional abuse. Emotional and physical neglect | PANSS | High DT+ showed higher scores on PANSS positive scale than DT-.  No significant differences in scores for PANSS negative scale.  Effect size (η2 = 0.10) | PANSS | High DT+ showed higher scores on measure of excitement compared to DT-  No difference in scores between groups for disorganisation and emotional distress |
| Davidson, Shannon, Mulholland, Campbell  (2009) | Prospective cohort | 1b | 31 (17 DT+)  20-69  Clinical psychosis with SCZ, SAD  (ICD)  Medication information  n/r | CTQ    Emotional, physical, sexual abuse, emotional, and physical neglect | Revised Manchester KGVM: positive and negative symptoms | No significant differences between DT+ and DT- on measures of psychosis (positive and negative symptoms) | KGVM: | No significant differences between DT+ and DT- on measures of affective states. |
| Lysaker, Beattie, Strasburger, Davis  (2005) | Prospective cohort | 1b | 65 (18 DT+)  24-65  Clinical psychosis with SCZ, SAD (DSM-IV)  Medication information  n/r  Pt with current substance abuse were excluded.  No changes in medication 1 month before study | CEQ  Sexual abuse | PANSS, WCST, DS | DT+ scored higher on PANSS positive than DT-.  DT+ scored lower on executive function measure than DT- (WCST).  No differences between groups in visuomotor processing speed (DS)  No overall time effects were found for any PANSS score | PANSS | DT+ scored higher on PANSS emotional discomfort than DT- (depressed mood and anxiety) |
| Lysaker, Meyer, Evans, Marks (2001) | Cross-sectional | 2b | 43 (15 DT+)  24-62  Clinical psychosis with SCZ, SAD (DSM-IV)  Medication information  n/r  No changes in medication 1 month before study | Self-report questionnaire    Sexual abuse (adult exposing, touching genitals, intercourse with adult) | PANSS; WCST; LNS; CVLT; DS; Vocabulary subset of the WAIS III | DT+ scored higher on PANSS positive and PANSS cognitive scale than DT-. No significant differences in scores for PANSS negative.  PANSS Positive:  DT+ 21.5 (5.7)  DT- 17.7 (4.5)  PANSS Negative:  DT+: 18.5 (5.9)  DT-: 16.7 (7.1)  PANSS Cognitive:  DT+: 20.3 (5.1)  DT- 16.0 (6.4    DT+ scored lower on working memory tests (WCST and LNS) and information processing (DS) than DT-. No differences in verbal memory scores (CVLT) | n/a | n/a |
| McCabe et al. (2012) | Case-control | 3b | 675 (408 SCZ, 267 HC)  18-65  Healthy controls and cinical psychosis-SCZ (ICD-10)  Medication information  n/r | CAQ    Sexual abuse, neglectful, Abusive, dysfunctional parenting loss, poverty, sibling loss | SANS; WTAR; WASI | Positive symptom severity was associated with greater number of experiences of DT  No associations between negative symptom severity and DT were found    DT+ in HC was associated with lower scores on IQ measures (WTAR) but not in P+DT | IPDE  GAF: functioning | High DT in P+DT and HC+DT was associated with personality clusters A, B and C |
| Shannon et al. (2011) | Cross-sectional | 2b | 85 (38 DT+)  41.1 (11.7)  Chronic SCZ  (DSM-IV)  Medication information  n/r | CTQ    Emotional, physical, sexual abuse, emotional and physical neglect | NART; WMS-III | DT+ scored lower on cognitive measures assessing working episodic memory compared to DT-    NART significant group differences (η2 = 0.09) | PDS; BDI: depression | DT+ scored higher on depression measures compared to DT-. (partial η2 = 0.05) |
| Aas et al. 2017 | Cross-sectional | 2b | 101 (48 with high DT+)  18-65  SCZ, SAD, schizophreniform disorder , ‘other’ psychotic disorders,  (SCID-I)  “Antipsychotics”, “Antidepressant” and  “Mood Stablisers” not specified | CTQ  (Low DT+ = ⩽41; High DT+ = >41)  Emotional, physical and sexual abuse, physical and emotional neglect | PANSS; experimental paradigm (positive/negative face recognition) | High DT+ have higher scores on positive symptoms measure than low DT+ (PANSS)    Experimental paradigm:  Increased brain area activation in response to negative faces in high DT+ compared to low DT-(Cohen's d: 0.72–0.77) | IDS; depression  GAF: functioning | High DT+ have higher scores on depression measure than low DT+  High DT+ scored lower on measures |
| Chatziioannidis et al. (2019) | Case-control | 3b | 63 (42 DT+)  SSP (DSM-IV and MINI)  Patients: 40.44 (10.003)  Controls: 39.33 (9.621)  “Antipsychotics” not specified in all patients at assessment  Pt with current substance related disorder were excluded. | CECA-Q  Mother antipathy/ neglect, father antipathy/neglect, physical abuse,  mother physical abuse, father physical abuse, and sexual abuse | PANSS | DT+ scored higher on PANSS P3 item (hallucinations) than DT-  r = 0 .375  Effect size n/r  Also, more hallucinations (P3 score) for MA+ compared to MA-, and MN+ compared to MN-  r = 0.338  Effect size n/r  No significant differences between DT+ and DT-, with mother antipathy DT and without, or with mother neglect DT and without (p<0.05) for cognitive factors on the PANSS  Effect size n/r | n/r | n/r |
| Comacchio et al. (2019) | Cross-sectional | 2b | 103 (physical DT+) 53 (sexual DT+) 234 (physical DT-) 274 (sexual DT-)  18-54  FEP  Medication information  n/r  Pt with antipsychotic prescription >3 months were excluded | CECA-Q  Physical abuse, and sexual abuse | PANSS | Physical DT+ higher negative symptoms than physical DT-  No difference between sexual DT+ and DT-  Effect size n/r | Age at psychosis onset | Females: 33.4 (10.2)  Males: 27.9 (8.5)  Physical and sexual DT+ decreases the age at onset for females only, compared to physical and sexual DT-  Effect size n/r |
| Cui et al. (2019) | Cross-sectional | 2b | 314 (159 with high DT+)  18-45  SSDs, delusional disorder, brief psychotic disorder (DSM-5)  Medication information  n/r | ETISR-SF  General trauma, physical punishment, emotional and sexual abuse | PANSS | High DT+ showed significantly lower scores on PANSS negative scale than DT-  No significant differences in scores for positive symptoms. | C-SSRS: suicidal ideation  CDSS: depression BCSS: schemata; BES; BS | High DT+ had more suicidal ideation (C-SSRS), lower scores for positive schema/higher scores for negative schema (BCSS), higher degree of rumination  about past negative events and were more depressed than low DT+  Effect size (C-SSRS): n/r  Effect size (BCSS): 0.502 (negative-self), 0.238 (positive-self), 0.722 (negative-others), 0.266 (positive-others)  Effect size (BS): 0.812(total)  Effect size (CDSS): 0.328 |
| Tosato et al. (2019) | Cross-sectional | 2b | 148 (95 DT+)  18-54  FEP (ICD-10)  Medication information  n/r  Pt with antipsychotic prescription >3 months were excluded | CECA-Q  Sexual abuse, physical abuse, separation and/or loss from parent figure(s) | PANSS; ICD-10 | No significant differences between DT+ and DT= on measures of psychosis (positive and negative symptoms)  Effect size n/r  No significant difference in psychosis diagnoses between DT+ and DT-  Effect size n/r | HAMD: depression  BRMS: mania | No significant differences between DT+ and DT- for depression (and mania scores    Effect sizes n/r |
| Anglin et al. (2019) | Cross-sectional | 2b | 816 (642 DT+)  18-29  PLE in the general Population  Medication information  n/r | TLE  Sexual or Physical Assault, Captivity, Combat or War Zone Exposure, Severe Human Suffering, Toxicity Exposure, Life-threatening Illness/Injury, Caused Serious Injury/Harm to Another, Fire or Explosion, Serious Accident, Transportation Accident, Sudden Death of Someone Close, Natural Disaster | PQ-Likert | There are significant effects on PLE for both the 1-3 and 4+ DT+ groups  The total effect of 4+ DT+ is larger than the total effect of 1–3 DT+ on PLE  Effect size n/r | DSS: Dissociation  ASI: | Correlations between PLE and dissociation differed significantly from zero with a dose response effect for DT+ participants (1-3 TLE and 4+ TLE)  Effect size n/r |
| Velikonja et al. (2019) | Cross-sectional | 2b | 225 (127 DT+)  18-64  SPD (DSM-IV (SCID-IV, SIDP-IV) and DSM-5 (SCID-5))  Medication information  n/r  Antipsychotics not an exclusion criterion if psychosis present despite prescription. | CTQ    Emotional, physical and sexual abuse, physical and emotional neglect | SPQ; MATRICS  Consensus Cognitive Battery | On average, more cognitive-perceptual symptoms in DT+ reporting severe forms of sexual abuse, but without showing statistical significance  No differences in the disorganized factor of SPQ between DT+ and DT-  DT+ reporting experiences of severe trauma performed significantly worse on most measures of working memory, verbal learning, visual learning and verbal fluency  Effect size: 0.66 (WMS Spatial Span, Forward), 0.90 (WMS Spatial Span, Backward), 1.45 (Letter Number Span, total), 1.27 (Letter Number Span, Longest Span), 0.88 (PASAT),  0.63 (HVLT),1.20 (BVMT), 0.79 (Category fluency), 1.62 (Executive functioning) | n/r | n/r |
| Boden et al. (2016) | Prospective cohort | 1b | 1018 (191 moderate DT+; 59 high DT+) participants with psychotic symptoms  followed up at 18, 21, 25, 30, 35  Medication information  n/r | Three-level scale; retrospective reports    Bullying victimization | Likert scale | High DT+ (bullying victimisation) group had 1.5-3 times higher rates of symptoms than DT-  Pooled means show a significant association between DT and psychosis symptoms  Effect size n/r | n/r | n/r |
| Schenkel et al. (2005) | Cross-sectional | 4 | 40 (18 DT+, 22 DT-) participants with SCZ; SAD (DSM-IV) aged 20-62  All pt were on atypical antipsychotics at time of testing | Social history interview  Childhood neglect, physical abuse and sexual abuse | BPRS; SILS; HSCT; BSAT; COWAT; CIT | No significant differences in BPRS factors between DT+ and DT-  DT+ with higher frequency and severity of abuse showed higher hallucinations and delusions than DT+ with less frequent and less severe abuse  No other BPRS factors show association with frequency or severity of abuse  Effect size n/r | BPRS: depression, anxiety | DT+ participants show higher scores on the anxiety/depression factor when compared to DT-.  Effect size n/r |
| Laskemoen et al. (2020) | Cross-sectional | 2b | 766 (365 DT+, 92 physical DT +,  122 sexual DT +, 208  emotional DT +, 209 emotional neglect, 167 physical neglect  M=30.9 (SD=10.6)  SSDs; BD (SCID)  “Antipsychotic”, “Anxiolytic/Hypnotic”, “Antidepressant”, “Antiepileptic” and/or “Lithium” status was recorded  Substance/alcohol misuse not an exclusion criterion | CTQ-SF  Emotional, physical and sexual abuse, physical and emotional neglect | PANSS | Childhood trauma associated with higher PANSS positive symptoms.  Effect size n/r | IDS-C: Depression  YMRS: mania | DT + reported higher frequency of insomnia than DT- (39%) (χ2 = 11.84, *p* = 0.001).  DT + reported lower frequency (26%) of hypersomnia than DT- (34%) (χ2 = 5.11, *p* < 0.05).  DT associated with depressive/anxiety symptoms of PANSS.  No differences in mania were observed.  Effect size n/r |
| Mørkved, et al. 2020 | Cross-sectional | 2b | 78 (41 DT+/37 DT)  With SSDs (ICD-10)  Average age: 29.8 (SD = 12.4)  “Antipsychotics” not specified | CTQ-SF  Emotional, physical and sexual abuse, physical and emotional neglect | PANSS  WAIS-III, D- KEFS, TMT, GPT, ROCFT, CVLT, CALCAP | DT+ had higher positive, negative and total PANSS symptoms than DT-.)  Physical neglect predicted impairment in global cognitive performance (*p* < .001), visuospatial abilities (*p* < .001), attention/ working memory (*p* < .001) and memory (*p* < .001).  Effect size n/r | n/r | n/r |
| Brañas et al (2022) | Cross-sectional | 4 | 62 (30 DT-, 23 physical or emotional abuse, 9 sexual abuse)  DT- : 30.41 (SD= 8.62)(  physical or emotional abuse: 32.26 (SD=8.45)  sexual abuse: 30.78 (SD=6.83)  SCZ, SAD, schizophreniform disorder psychotic bipolar disorder, psychotic depression, DD, PDNOS and brief psychotic disorder (DSM-IV)  Medication information  n/r | Semi-structured interview  Emotional neglect, psychological abuse, physical abuse, or sexual abuse) before age 16. | PANSS | No differences in positive, negative, general psychopathology or total PANSS score between sexual abuse, physical or emotional abuse, and no abuse groups  Effect size n/r | The Degraded Facial Affect Recognition Task (DFAR)  Hinting Task | No differences in emotion recognition of happy, angry, neutral faces, total correct answer  No differences in hinting task score  Physical/emotional abuse group reported greater fear recognition than sexual abuse and no abuse groups (*p* = 0.008)  More frequent fear misinterpretation as neutral in the sexual abuse group compared to the physical or emotional abuse group (*p* = 0.040)  Effect size n/r |
| Ayesa-Arriola et al. (2020) | Case- control | 3b | 342 (290 FEP patients and 52 HC)  15-65  FEP:30.46 (9.65)  HC: 8.25 (7.83)  “Antipsychotics” not specified  Exclusion of pt with antipsychotics > 6 weeks and substance or alcohol dependence | Childhood Traumatic Events Scale  death of close friend or family member; parents’ divorce; sexual abuse; physical abuse; sickness/accident; or other) | SANS-SAPS: positive, negative,  PAS, DAS, RAVLT, WAIS-III, TMT, GPT, CPT | No differences in positive, negative or disorganized symptoms between DT+, DT+ and adult trauma, DT- and adult trauma and DT- no adult trauma  DT+ and adult trauma showed worse working memory than DT-  DT+ had lower general cognitive function  Effect size n/r | CDSS: Depression  SANS-SAPS:  disorganize d symptoms | DT + and adult trauma had higher depressive scores than DT+, DT- and adult trauma and DT- no adult trauma  No differences in disorganized symptoms between DT+, DT+ and adult trauma, DT- and adult trauma and DT- no adult trauma  Effect size n/r |
| Kilian et al. (2020) | Prospective cohort study | 2b | 56 (34 high DT/ 22 low DT)  16 -45  FEP (DSM-IV criteria for schizophreniform disorder, schizophrenia or schizo-affective disorder  Medication information  n/r at baseline  Exclusion of pt with antipsychotics > 4 weeks | CTQ-SF  Emotional, physical and sexual abuse, physical and emotional neglect | PANSS | No differences in PANSS positive, negative or total scores between high and low DT.  Effect size n/r | CDSS: depression  PANSS disorganization: | High DT had higher CDSS total scores than low DT (*p* = 0.046)  High DT had lower global QOL scores than low DT (*p* = 0.002)  No differences in disorganization  Effect size n/r |
| Van dam et al. (2015) | Prospective controlled cohort study | 1b | 1119 Patients (44% high DT) , 1057 siblings (25% high DT) and 589 controls (19% high DT)  16-50  SCZ, schizophreniform disorder, SAD, delusional disorder, psychotic disorder NOS (DSM-IV)  Medication information  n/r | CTQ-SF  Emotional, physical and sexual abuse, physical and emotional neglect | PANSS (patients):  SIS-R (siblings, controls) | In patients, abuse showed a stronger association with positive symptoms than neglect (B 0.24, 95% CI 0.09–0.40, *p* = 0.002).  Abuse and neglect didn’t influence course of symptoms over 3 years on PANSS positive and negative symptoms in any group.  Neglect did not show any specificity for symptoms; there were no differences in influence of neglect between all three symptom clusters.  Effect size: Total trauma: -0.07 (Patients, positive vs. negative symptoms), -0.06 (Siblings, positive vs. negative schizotypy), -0.04 (Controls, positive vs. negative schizotypy). Abuse: -0.19 (Patients, positive vs. negative symptoms), -0.04 (Siblings, positive vs. negative schizotypy), -0.08 (Controls, positive vs. negative schizotypy). Neglect: 0.003 (Patients, positive vs. negative symptoms), -0.0003 (Siblings, positive vs. negative schizotypy), -0.03 (Controls, positive vs. negative schizotypy). | CDS (patients): depressive symptoms  CAPE(siblings, controls): depressive symptoms | Abuse was more strongly associated with depressive than with negative symptoms.  Abuse and neglect didn’t influence course of symptoms over 3 years  Abuse and neglect didn’t influence course of symptoms over 3 years on PANSS depressive symptoms in any group.  Effect size: Total trauma: (Positive symptoms/schizotypy vs. depressive symptoms: -0.06 (patients), 0.04 (siblings), 0.09 (controls)) (Negative symptoms/schizotypy vs. depressive symptoms: -0.04 (patients), -0.13 (siblings), -0.19 (controls)). Abuse: (Positive symptoms/schizotypy vs. depressive symptoms: -0.07 (patients), 0.09 (siblings), 0.06 (controls)) (Negative symptoms/schizotypy vs. depressive symptoms: -0.18 (patients), -0.16 (siblings), -0.16 (controls)). Neglect: (Positive symptoms/schizotypy vs. depressive symptoms: -0.01 (patients), 0.04 (siblings), 0.07 (controls)) (Negative symptoms/schizotypy vs. depressive symptoms: 0.05 (patients), -0.07 (siblings), -0.14 (controls)). |
| Bell et al. (2019) | Prospective cohort | 1b | 984 (80 low sexual DT+; 64 high sexual DT+; 110 low physical DT+; 66 high physical DT+)  18, 21, 25, 30, 35  With psychotic experience in adulthood (DSM-IV) | Three-level scales    Sexual abuse and physical abuse | Abnormal thought; abnormal perception | High sexual DT+ report greater psychotic experiences, in terms of levels of both abnormal thought and perception symptoms (from ages 25-30 and 30-35), than the low sexual DT+ and sexual DT- groups  Physical DT+ is associated with greater experiences of abnormal thought and perception than the physical DT- group | n/r | n/r |
| Read et al. (2003) | Cohort study (chart review) | 2b | 200 (60 DT+)  Aged 18-69  With  SCZ; SAD (DSM-IV), depression; substance abuse; BD; personality disorder; anxiety disorder; adjustment disorder; PTSD; psychotic episode; psychosis disorder | Medical records    Physical abuse and sexual abuse | Clinical diagnostic (medical records) following the ‘characteristic symptoms’ of schizophrenia listed in DSM-IV | Sexual DT+ participants show higher frequency and mean number of DSM-IV symptoms than DT- group  Hallucinations are more common in sexual and/or physical DT+ than DT-.  No differences in delusions, thought disorder or negative symptoms between DT+ and DT-  Physical and/or sexual DT+ more commonly reported different sensory modalities of hallucinations than DT-  No differences were observed in the different types of delusions between DT+ and DT- | n/r | n/r |

| Table 2. Characteristics of Qualitative Studies | | | | | | |  |  |
| --- | --- | --- | --- | --- | --- | --- | --- | --- |
| **Literature** | **Design** | **Evidence**  **level** | **Sample (N of groups, age range or M/SD, diagnoses, medication)** | **Measure of DT and types of DT** | **Primary outcome**  **measure** | **Primary outcome** | **Secondary outcome measure** | **Secondary outcomes** |
| Misiak et al. (2016) | Cross-sectional | 2b | 94 (37 DT+)  FES  (DSM-IV and ICD-10)  Male:  24.81 (4.6)  Female:  28.62 (6.08) | ETISR-SF    General trauma, physical punishment, emotional and sexual abuse | OPCRIT checklist | Third person auditory hallucinations, abusive/accusatory/persecutory voices and first-rank hallucinations were more frequent in FES with DT+ | n/r | n/r |
| Dorahy et al. (2009) | Cross-sectional | 2b | 65 (16 DT+)  19-63  SCZ (DSM-IV-TR) | CTQ    Emotional, physical and sexual abuse, physical and emotional neglect | MUPS | Prevalence and phenomenology of auditory hallucinations, such as command hallucinations, and hallucinations in other modalities differed between DT+ and DT- | DES-T | DT+ scored higher on a dissociative experiences measure than DT- |
| Offen et al. (2003) | Cross-sectional | 2b | 26 (10 DT+)  23-67  SCZ; schizoid, psychotic depression, psychosis  (DSM-IV) | Self-reported questionnaire    Sexual abuse | BAVQ | No differences in the perceived nature of the hallucinations (benevolence, resistance, engagement, power) between DT+ and DT-  DT+ more commonly reported malevolent hallucinations than DT- after adjusting for age | DES-II; DES-T; BDI | DT+ scored higher on dissociation (DES-II; DES-T) and depression (BDI) scales than DT- |
| Hardy et al. (2005) | Cross-sectional | 2b | 75 (7 DT+)  18-65  Hallucinations in individuals with SCZ; SAD;  (SAPS) | Trauma history questionnaire    Sexual abuse | Schedules for Clinical Assessment in Neuropsychiatry; PSRS | Evidence of indirect (thematic) associations between hallucinatory content and DT | n/r | n/r |
| Reiff et al. (2012) | Cross-sectional | 2b | 30 (22 DT+)  18-65  Hallucinations and delusions in individuals with SCZ, SAD, and BD  (DSM–IV–TR) | Semi-structured interview  Physical abuse (hitting, kicking, burning, stabbing, shooting) | PRISM; SCID | Hallucinatory content was associated with DT characteristics. Threatening & fear-invoking hallucinations, hallucinations in different modalities, malevolent voices and voices relating to a known person were more common in DT+ than DT- | n/r | n/r |
| Begemann et al., 2021 | Case-control | 2b | 413  Low DT: 299  Emotion-focused DT:71  High DT: 43  Aged 18-65 with AVH, SCZ, SAD, psychosis not otherwise specified | CTQ-SF  Emotional, physical and sexual abuse, physical and emotional neglect | PSRS | High DT + had greater amount of negative content compared to low DT +  High DT+ had higher controllability than emotional DT+  No differences in frequency, duration, location, loudness, belief about origin, degree and amount of distress. impact on functioning or number of voices between high and low DT+ | BAVQ | High DT+ rated their voices as more malevolent and felt more resistance against them compared to low DT+  High DT+ felt auditory  verbal hallucination as  more omnipotent than  low DT+ (p = 0.021). |

Footnote: AVH: Auditory Verbal Hallucinations ASI: Aberrant Salience Inventory; BAVQ: Beliefs About Voices Questionnaire; BCSS: Brief Core Schema Scales; BD: Bipolar Disorder; BDI/BDI-II: Beck Depression Inventory; BDRS: Bipolar Depression Rating Scale; BES: Basic Empathy Scale; BPRS: Brief Psychiatric Rating Scale; BRMS: Bech-Rafaelsen Mania Rating Scale; BS: Brooding Scale; BSAT: The Brixton Spatial Anticipation Test; BSI: Brief Symptom Inventory; BSSI: Beck Scale for Suicide Ideation; C-SSRS: Columbia Suicide Severity Rating Scale; CAQ: Childhood Adversity Questionnaire; CDS: Calgary depression scale; CDSS: Calgary Depression Scale for Schizophrenia. CECA-Q: Childhood Experience of Care and Abuse-Questionnaire; CEQ: Childhood Experiences Questionnaire; CHQ: Childhood History Questionnaire; CIT: The Contour Integration Test; COWAT: Controlled Oral Word Association Test; CTQ/ CTQ-SF: Childhood trauma questionnaire (short-form); CTES: Childhood Traumatic Events Scale; CVLT: California Verbal Learning test; DAS: Disability Adjustment Scale; DASS-21: The Depression, Anxiety and Stress Scale - 21 Items; DES-II: Dissociative Experiences Scale; DES-T: Dissociative Experiences Scale-Taxon; DIS: Diagnostic Interview Schedule; DS: Digit Symbol subset of the WAIS III; DSM-IV-TR: Diagnostic and Statistical Manual of Mental Disorders, 4th Edition, Text Revision; DSS: Dissociative Symptoms Scale; DT+: with DT history; DT-: without DT history; ETISR-SF: Early Trauma Inventory Self-Report — Short Form; FEP: First-episode psychosis; FES: First-episode schizophrenia; GAF: Global assessment of function; GSI: General severity index; HAMD: Hamilton Rating Scale for Depression; HC: Healthy control; HC+DT/HC-DT: Healthy control with DT/without DT; HSCT: The Hayling Sentence Completion Test; ICD: International classification of disease; IDS: Inventory of Depressive Symptoms; IDS-C: Inventory of Depressive Symptomatology-Clinician version; IPDE: International Personality Disorder Examination; IQ: Intelligence Quotient; KGVM: Krawiecka Goldberg Vaughan–Modified Symptom Scale; LNS: Letter Number sequencing subtest of the WAIS III; LEC: Life-events checklist; MACE: Maltreatment and Abuse Chronology of Exposure scale; MA+/MA-: With/Without mother antipathy; MADRS: Montgomery-Åsberg Depression Rating Scale; MINI: Mini-International Neuropsychiatric Interview 5.0.0; MN+/MN-: With/Without mother neglect; MUPS: Mental Health Research Institute Unusual Perceptions Schedule; n/a: Not available; n/r: Not reported; NART: National Adult Reading Test; OPCRIT: Operational Criteria for Psychotic Illness checklist; PANSS: Positive and Negative Syndrome Scale; PAS: premorbid adjustment scale; PDS: Posttraumatic diagnostic scale; P+DT/P-DT: Psychosis with DT/psychosis without DT; PLE: Psychotic-like experiences; PQ-Likert: Prodromal Questionnaire-Likert; PRISM: Psychiatric Research Interview for Substance and Mental Disorders; PSRS: Psychotic Symptom Rating Scale; PTSD: Post-Traumatic Stress Disorder; RBANS: Repeatable battery of the assessment of neuropsychological status; SAD: Schizoaffective disorder; SANS: Scale for the Assessment of Negative Symptoms; SAPS: Scale for the Assessment of Positive Symptoms; SCID/SCID-I: Structured Clinical Interview for DSM-IV Axis I Disorders; SCL-90: Symptom Checklist 90; SCZ: Schizophrenia; SIDP-IV: Structured Interview for DSM-IV Personality; SILS: The Shipley Institute of Living Scale; SPD: Schizotypal personality disorder; SPQ: Schizotypal Personality Questionnaire; SSDs: Schizophrenia Spectrum Disorders; SSP: Schizophrenia-spectrum psychosis; TLE: Traumatic life events; WAIS III: Wechsler Adult Intelligence Scale-III; WASI: Wechsler Abbreviated Scale of Intelligence; WCST: Wisconsin Card Sorting Test; WMS-III: Wechsler Memory Scale-Third Edition; WTAR: Wechsler Test of Adult Reading; YMRS: Young Mania Rating Scale

**List of all included papers**

1. McCabe KL, Maloney EA, Stain HJ, Loughland CM, Carr VJ. Relationship between childhood adversity and clinical and cognitive features in schizophrenia. *J Psychiatr Res*. 2012;46(5):600-607. doi:10.1016/j.jpsychires.2012.01.023

1. [Ayesa-Arriola R, Setiên-Suero E, Marques-Feixa L, et al. The synergetic effect of childhood trauma and recent stressful events in psychosis: associated neurocognitive dysfunction.](#ayesaarriola2020) *[Acta Psychiatr Scand.](#ayesaarriola2020)* [2020;141(1):43-51. doi: 10.1111/acps.13114.](#ayesaarriola2020)

1. [Quidé Y, O’Reilly N, Watkeys OJ, Carr VJ, Green MJ. Effects of childhood trauma on left inferior frontal gyrus function during response inhibition across psychotic disorders.](#quide2018) *[Psychol Med](#quide2018)*[. 2018;48(9):1454-1463. doi:10.1017/S0033291717002884](#quide2018)
2. [Schalinski I, Breinlinger S, Hirt V, Teicher MH, Odenwald M, Rockstroh B. Environmental adversities and psychotic symptoms: The impact of timing of trauma, abuse, and neglect.](#schalinski2019) *[Schizophr Res](#schalinski2019)*[. 2019;205:4-9. doi:10.1016/j.schres.2017.10.034](#schalinski2019)

1. [Schalinski I, Fischer Y, Rockstroh B. Impact of childhood adversities on the short-term course of illness in psychotic spectrum disorders.](#schalinski2015) *[Psychiatry Res](#schalinski2015)*[. 2015;228(3):633-640. doi:10.1016/j.psychres.2015.04.052](#schalinski2015)

1. [Chatziioannidis S, Andreou C, Agorastos A, et al. The role of attachment anxiety in the relationship between childhood trauma and schizophrenia-spectrum psychosis.](#chatziioannidis2019) *[Psychiatry Res](#chatziioannidis2019)*[. 2019;276:223-231. doi:10.1016/j.psychres.2019.05.021](#chatziioannidis2019)

1. [Mohammadzadeh, A., Azadi, S., King, S., Khosravani, V., & Bastan, F. S. (2019). Childhood trauma and the likelihood of increased suicidal risk in schizophrenia.](#mohammadzadeh2019) *[Psychiatry Research,](#mohammadzadeh2019)* [Volume 275, 100-107. doi:](#mohammadzadeh2019) <https://doi.org/10.1016/j.psychres.2019.03.023>
2. Lamela D, Figueiredo B. Childhood physical maltreatment with physical injuries is associated with higher adult psychopathology symptoms. *Eur Psychiatry*. 2018;53:1-6. doi:10.1016/j.eurpsy.2018.04.008

1. [Misiak B, Moustafa AA, Kiejna A, Frydecka D. Childhood traumatic events and types of auditory verbal hallucinations in first-episode schizophrenia patients.](#misiak2016) *[Compr Psychiatry](#misiak2016)*[. 2016;66:17-22. doi:10.1016/j.comppsych.2015.12.003](#misiak2016)

1. [Kelly DL, Rowland LM, Patchan KM, et al. Schizophrenia clinical symptom differences in women vs. men with and without a history of childhood physical abuse.](#kelly2016) *[Child Adolesc Psychiatry Ment Health](#kelly2016)*[. 2016;10(1):5. doi:10.1186/s13034-016-0092-9](#kelly2016)
2. [Lysaker PH, Meyer P, Evans JD, Marks KA. Neurocognitive and symptom correlates of self-reported childhood sexual abuse in schizophrenia spectrum disorders.](#lysaker2001) *[Ann Clin Psychiatry](#lysaker2001)*[. 2001;13(2):89-92. doi:10.1023/A:1016667624487](#lysaker2001)

1. [Shannon C, Douse K, McCusker C, Feeney L, Barrett S, Mulholland C. The Association Between Childhood Trauma and Memory Functioning in Schizophrenia.](#shannon2011) *[Schizophr Bull](#shannon2011)*[. 2011;37(3):531-537. doi:10.1093/schbul/sbp096](#shannon2011)
2. Aas M, Kauppi K, Brandt CL, et al. Childhood trauma is associated with increased brain responses to emotionally negative as compared with positive faces in patients with psychotic disorders. *Psychol Med*. 2017;47(4):669-679. doi:10.1017/S0033291716002762
3. Dorahy MJ, Shannon C, Seagar L, et al. Auditory hallucinations in dissociative identity disorder and schizophrenia with and without a childhood trauma history: Similarities and differences. *J Nerv Ment Dis*. 2009;197(12):892-898. doi:10.1097/NMD.0b013e3181c299ea
4. Offen L, Waller G, Thomas G. Is reported childhood sexual abuse associated with the psychopathological characteristics of patients who experience auditory hallucinations? *Child Abuse Negl*. 2003;27(8):919-927. doi:10.1016/S0145-2134(03)00139-X
5. Hardy A, Fowler D, Freeman D, et al. Trauma and hallucinatory experience in psychosis. *J Nerv Ment Dis*. 2005;193(8):501-507. doi:10.1097/01.nmd.0000172480.56308.21
6. Anglin DM, Espinosa A, Barada B, et al. Comparing the Role of Aberrant Salience and Dissociation in the Relation between Cumulative Traumatic Life Events and Psychotic-Like Experiences in a Multi-Ethnic Sample. *J Clin Med*. 2019;8(8):1223. doi:10.3390/jcm8081223

1. [Schenkel LS, Spaulding WD, Dilillo D, Silverstein SM. Histories of childhood maltreatment in schizophrenia: Relationships with premorbid functioning, symptomatology, and cognitive deficits.](#schenkel2005) *[Schizophr Res](#schenkel2005)*[. 2005;76:273-286. doi:10.1016/j.schres.2005.03.003](#schenkel2005)

1. [Tosato S, Bonetto C, Tomassi S, et al. Childhood trauma and glucose metabolism in patients with first-episode psychosis.](#tosato2020) *[Psychoneuroendocrinology](#tosato2020)*[. 2020;113. doi:10.1016/j.psyneuen.2019.104536](#tosato2020)
2. Velikonja T, Velthorst E, McClure MM, et al. Severe childhood trauma and clinical and neurocognitive features in schizotypal personality disorder. *Acta Psychiatr Scand*. 2019;140(1):50-64. doi:10.1111/acps.13032

1. [Cui Y, Kim SW, Lee BJ, et al. Negative schema and rumination as mediators of the relationship between childhood trauma and recent suicidal ideation in patients with early psychosis.](#cui2019) *[J Clin Psychiatry](#cui2019)*[. 2019;80(3). doi:10.4088/JCP.17m12088](#cui2019)

1. [Comacchio C, Howard L, Bonetto C, et al. The impact of gender and childhood abuse on age of psychosis onset, psychopathology and needs for care in psychosis patients.](#comacchio2019) *[Schizophr Res](#comacchio2019)*[. 2019;210:164-171. doi:10.1016/j.schres.2018.12.046](#comacchio2019)

1. [Read J, Agar K, Argyle N, Aderhold V. Sexual and physical abuse during childhood and adulthood as predictors of hallucinations, delusions and thought disorder.](#read2003) *[Psychol Psychother Theory, Res Pract](#read2003)*[. 2003;76:1-22. doi:10.1348/14760830260569210](#read2003)

1. [Laskemoen JF, Aas M, Vaskinn A, et al. Sleep disturbance mediates the link between childhood trauma and clinical outcome in severe mental disorders.](#laskemoen2021) *[Psychol Med](#laskemoen2021)*[. 2021;51(14):2337-2346. doi: 10.1017/S0033291720000914](#laskemoen2021)
2. Begemann MJH, Sommer IE, Brand RM, et al. Auditory verbal hallucinations and childhood trauma subtypes across the psychosis continuum: a cluster analysis. *Cogn Neuropsychiatry*. 2021;27(2-3):150-168. doi: 10.1080/13546805.2021.1925235

1. [Mørkved N, Johnsen E, Kroken RA, et al. Does childhood trauma influence cognitive functioning in schizophrenia? The association of childhood trauma and cognition in schizophrenia spectrum disorders. Schizophr Res Cogn. 2020;19;21:100179. doi: 10.1016/j.sc](#morkved2020)
2. Brañas A, Lahera G, Barrigón ML, Canal-Rivero M, Ruiz-Veguilla M. Effects of childhood trauma on facial recognition of fear in psychosis. *Rev Psiquiatr Salud Ment (Engl Ed).* 2019;11;S1888-9891(19)30022-9. doi: 10.1016/j.rpsm.2019.01.005.

1. [Davidson G, Shannon C, Mulholland C, Campbell J. A longitudinal study of the effects of childhood trauma on symptoms and functioning of people with severe mental health problems.](#davidson2009) *[J Trauma Dissociation](#davidson2009)*[. 2009;10(1):57-68. doi:10.1080/15299730802485169](#davidson2009)

1. [Lysaker PH, Beattie NL, Strasburger AM, Davis LW. Reported History of Child Sexual Abuse in Schizophrenia.](#lysaker2005) *[J Nerv Ment Dis](#lysaker2005)*[. 2005;193(12):790-795. doi:10.1097/01.nmd.0000188970.11916.76](#lysaker2005)
2. Boden JM, Van Stockum S, Horwood LJ, Fergusson DM. Bullying victimization in adolescence and psychotic symptomatology in adulthood: Evidence from a 35-year study. *Psychol Med*. 2016;46(6):1311-1320. doi:10.1017/S0033291715002962
3. Bell CJ, Foulds JA, Horwood LJ, Mulder RT, Boden JM. Childhood abuse and psychotic experiences in adulthood: Findings from a 35-year longitudinal study. *Br J Psychiatry*. 2019;214(3):153-158. doi:10.1192/bjp.2018.264
4. van Dam DS, Nierop Mv, Viechtbauer W, et al. Childhood abuse and neglect in relation to the presence and persistence of psychotic and depressive symptomatology. *Psych Med.* 2015;45:1363-1377. doi: 10.1017/S0033291714001561
5. Kilian S, Asmal L, Chiliza B, et al. T4. Childhood Trauma And Its Relationship With Premorbid Adjustment, Cognition And Treatment Outcomes In First Episode Schizophrenia. *Schizophr Bull*. 2019;45(Supplement_2):S204-S205. doi:10.1093/schbul/sbz019.284
6. Reiff M, Castille DM, Muenzenmaier K, Link B. Childhood abuse and the content of adult psychotic symptoms. *Psychol Trauma Theory, Res Pract Policy*. 2012;4(4):356-369. doi:10.1037/a0024203
